# Quality of care in psychiatry is related to research activity

**DOI:** 10.1192/j.eurpsy.2021.16

**Published:** 2021-03-04

**Authors:** J. de Arriba-Enriquez, E. Sanz-Casado, E. Vieta, M. Rapado-Castro, C. Arango

**Affiliations:** 1 CIBERNED, Network Centre for Biomedical Research in Neurodegenerative Diseases, National Institute of Health Carlos III, Madrid, Spain; 2 Research Institute for Higher Education and Science (INAECU), University Carlos III of Madrid and Autonomous University of Madrid, Madrid, Spain; 3 Hospital Clinic, Institute of Neuroscience, University of Barcelona, IDIBAPS, CIBERSAM, Barcelona, Catalonia, Spain; 4 Department of Child and Adolescent Psychiatry, Institute of Psychiatry and Mental Health, Hospital General Universitario Gregorio Marañón, School of Medicine, Universidad Complutense, IiSGM, CIBERSAM, Madrid, Spain; 5 Melbourne Neuropsychiatry Centre, Department of Psychiatry, The University of Melbourne & Melbourne Health, Melbourne, VI, Australia

**Keywords:** Bibliometrics, biomedical research, healthcare quality, mental health, psychiatry

## Abstract

**Background:**

The top biomedical research institutions have traditionally been assumed to provide better medical treatment for their patients. However, this may not necessarily be the case. Low-to-moderate negative associations between research activity and the quality-of-care provided by clinical departments have been described. We aimed to examine this relationship in the psychiatric units of the largest hospitals in Spain.

**Methods:**

Scientific publications for 50 hospitals were retrieved from the Web of Science (2006–2015), and quality of mental healthcare data were gathered from Spanish National Health System records (2008–2014). Spearman-rank correlation analyses (adjusting for number of beds and population) were used to examine the associations between research data and quality-of-care outcomes in psychiatry. Stepwise regression models were built in order to determine the predictive value of research productivity for healthcare outcomes.

**Results:**

We found a positive association between research activity indicators (i.e., number of publications, number of citations, cumulative impact factor, and institutional H-index) and better quality-of-care outcomes in psychiatry (i.e., number of readmissions, transfers, and discharges from hospital). In particular, a higher research activity predicted a lower level of readmissions for individuals with psychoses (*p* = 0.025; *R* = 0.317), explaining 8.2% of the variance when other factors were accounted for.

**Conclusions:**

Higher research activity is associated with better quality of mental healthcare in psychiatry. Our results can inform decision-making in clinical and research management settings in order to determine the most appropriate quality measures of the impact of research on the prognosis of individuals with psychiatric conditions.

## Introduction

It is commonly asserted that leading biomedical research hospitals provide better clinical care for their patients. However, there is no specific data to support this claim. Very few studies have analyzed the relationship between research productivity and quality of the healthcare provided in clinical research centers. Previous studies have assessed quality of care mostly in teaching hospitals [1]. These assessed the reputation of hospitals based on their level of scientific production [2] or explored the relationship between the number of publications and measures of research activity such as number of citations, scholarship selection processes, and investment in research and quality-of-care indicators [3,4].

A global study in England exploring the association between mortality rates in National Health Service (NHS) trusts and academic output (number of citations per admission) attributed to each NHS trust and constituent hospitals found a significant correlation between number of citations and mortality rates [5]. In cardiology, Pons et al. [6] studied the association between bibliometric measures of research outputs in hospitals in Spain using hospital mortality from two common cardiac conditions as an outcome measure. These conditions were congestive heart failure and acute myocardial infarction. This cross-sectional study established a low to moderate negative correlation between the mortality rate and the weighted citations ratio for both heart conditions, concluding that it was important to further research the interaction between research outcomes and clinical practice. In a different study, Lascurain et al. [7] descriptively assessed the impact of scientific production in the most frequently researched medical specialties (measured in terms of publications cited in MEDLINE) on the Spanish health system (in terms of R&D resources allocated to medical science, mortality, morbidity, and drug spending) using National Institute of Health data gathered between 1991 and 2002. Medical specialties were sorted by frequency of appearance using “The Serials Directory/EBSCO-CD-ROM” classification, with neuroscience being among the most frequently researched. In general, authors found a disparity between the causes of mortality in Spain and the subject matter of research papers published in Medline by Spanish institutions (both universities and healthcare centers). However, they observed an equivalence between the published research subject and the cause of morbidity. A similar pattern was identified in the analysis of drug spending, with neuroscience-related disorders, as the item occasioning the highest drug spending, being the medical area accounting for the largest share of papers published by Spanish researchers.

The lack of psychiatry-specific studies led us to explore the association between research and healthcare quality measures in this field. The current study aims to determine if there is an association between research activity and quality of care as provided in psychiatric units in Spanish hospitals. Spain has a public healthcare system, guaranteeing universal coverage for all residents. State healthcare is free of charge to anyone living and working in Spain. Around 90% of Spaniards use the public healthcare system, which is called the National Health System. The health system in Spain is very decentralized with service delivery organized at the regional level (Autonomous Communities). The system is overseen by the Spanish Ministry of Health, which develops policy and oversees the national health budget [8]. We hypothesized that there would be a positive correlation between research and healthcare quality. This would have important implications for clinical and research decision-makers, as it may help better determine the quality measures or indicators most appropriate for assessing the impact of research on the prognosis of individuals with mental health conditions.

## Methods

This is a national, longitudinal, descriptive study on the relationship between scientific production and mental healthcare outcomes over time carried out in the top 50 leading hospitals with psychiatric units in Spain. Selection, search, retrieval, gathering, and analysis of representative data indicators were carried out between the years 2008 and 2014 for healthcare activity and between 2006 and 2015 for research activity.

### Quality-of-care outcomes

The clinical research hospitals from which the data was retrieved were selected according to the following inclusion criteria: (a) being one of the following types of institution: hospital consortiums, teaching hospitals, or general hospitals, (b) being a public and/or private institution with a welfare approach, (c) having a psychiatric unit, (d) having data included in the Spanish National Health System records, (e) having more than 500 beds, and (f) serving a population of more than 50,000 inhabitants. These criteria were established based on available representative data gathered from the Spanish National Catalogue of Hospitals and the National Statistics Institute. We included hospitals with more than 500 beds/serving a population of more than 50,000 inhabitants. These are healthcare institutions with specialized units that receive referrals from other (smaller) clinical centers in the same area and are thus a representative sample of the population in their corresponding autonomous community in Spain. Healthcare outcome data were obtained from the Minimum Hospital Data Set (CMBD) for hospital discharge records and specialized outpatient care data collected from the National Health System (CMBD) of the Spanish Ministry of Health. CMBD data were obtained through an extraction request form submitted to the Spanish Health Information Institute, which provided data on patients with a main or predefined secondary diagnosis of a mental health condition.

Mental health diagnoses were selected on the basis of the most prevalent psychiatric disorders according to the International Classification of Diseases ICD-9-CM, as this was the classification used in the national database used. The a priori designated most prevalent diagnoses were grouped into eight different diagnostic groups by one of the expert coauthors (CA) based on his clinical and research experience in psychiatry. Consensus was reached with an expert external clinical psychiatrist who independently reviewed the selected diagnosis. Diagnoses were then grouped and analyzed as separate individual diagnoses as follows: (a) nonaffective psychosis disorders (schizophrenic disorders, delusional disorders, and other nonorganic psychoses), (b) bipolar disorders (bipolar I disorder, single manic episode, manic disorder recurrent episode, bipolar I disorder, most recent episode (or current) manic, bipolar I disorder, most recent episode (or current) depressed, bipolar I disorder, most recent episode (or current) mixed, bipolar I disorder, most recent episode (or current) unspecified, other and unspecified bipolar disorders, episodic mood disorders), (c) depressive disorders (depressive type psychosis, major depressive disorder single episode, major depressive disorder recurrent episode), (d) other affective disorders (cyclothymic disorder, dysthymic disorder, depressive disorder, not elsewhere classified), (e) personality disorders, (f) alcohol and drug abuse disorders (alcohol dependence syndrome, drug dependence, nondependent abuse of drugs, alcohol-induced mental disorders, and drug-induced mental disorders), (g) other organic mental disorders (persistent mental disorders due to conditions classified elsewhere), and (h) developmental disorders (other specified intellectual disabilities and unspecified intellectual disabilities).

Based on the healthcare delivery data provided by each of the participating clinical research institutions, the following measures were calculated for the 2008–2014 period: Average hospital stay, admissions (planned or unplanned), hospital discharges (voluntary discharge or discharge to home, discharges by psychiatric diagnoses, referrals, and exitus), and readmissions (readmission rate and nonreadmissions by psychiatric diagnoses within 30 days after discharge).

### Research activity outputs

Research outcome data were obtained by selecting psychiatry publications from the Web of Science-WoS (Clarivate Analytics) through the institutional affiliation of each of the participating clinical research centers. Psychology publications were excluded from this search due to the representativeness of the psychiatry area, the higher predominance of psychiatric professionals in the psychiatric units participating in this study, and the difficulty of distinguishing between publications on clinical and nonclinical psychology.

The scientific publication data retrieval was carried out through a personalized search strategy that included all possible affiliations or institutional names that the authors may have included in their publications (see supplementary material). All plausible affiliations were included in Spanish, English, and the official languages in Spain other than Spanish (i.e., Basque, Galician, and Catalonian) of the Autonomous Communities of each clinical center. In order to make the scientific activity retrieval process more precise, an additional search strategy (see supplementary material) was used in order to further compile the production of clinical research groups that are part of the Spanish Network for Biomedical Research in Mental Health (CIBERSAM) [9]. The search, filtering, and retrieving of bibliographic information was conducted by a data management and quality-of-care specialist.

A total of 3,888 records were obtained. Data was exported to EndNote bibliographic citation manager for editing. Automatic elimination of duplicates and a manual review of the records were peer-conducted by the data management specialist and a clinical psychiatrist. Research publications that did not belong to the participating centers or to the current study’s area of interest were excluded. A total of 2,263 valid documents were analyzed.

Data were exported to Microsoft Excel (Microsoft Corp., Redmond, WA) spreadsheets where indicators of scientific activity were calculated. Research indicators of the scientific activity of the clinical centers included: (a) Research production (proportion of publications produced in psychiatry during the 2006–2015 period by each center selected. Only original articles, reviews, and editorials as a typology were included), (b) Number of citations (number of citations during the 2006–2015 period, including self-citations), (c) Ratio of citations per documents (average number of citations per document including self-citations), (d) National collaborations (proportion of documents including only national CAs or institutions), (e) International collaborations (proportion of documents including at least one foreign CA or institution), (f) Impact factor (average and cumulative impact factor of publications produced by the analyzed institutions during the 2006–2015 period including self-citations), and (g) Institutional H-Index (the largest number *h* such that at least *h* articles from that institution were cited at least *h* times each).

### Statistical analysis

Normal distribution of variables was assessed by means of the Kolmogorov–Smirnov test. Means and standard deviations were used to describe continuous variables. Frequencies were used to describe discrete variables. All variables were transformed into ranges to adjust to a normal distribution. The proportion (relative frequency) expressed as a percentage was calculated for all variables of interest (i.e., research activity and quality of care variables) in order to establish their relative frequency. Spearman Rank Correlation analyses (controlling for number of beds and population, as appropriate) were used to explore the associations between research activity and quality-of-care variables.

In order to analyze the predictive value of research productivity (independent variables) for quality-of-care outcomes (dependent variables), stepwise regression models were built for those measures that showed significant associations in the previous Spearman’s Rho correlation tests (*R*_s_), adjusting for the number of beds and population (i.e., number of inhabitants) as variables of noninterest. All statistical analyses were performed using IBM SPSS Statistics for Windows, Version 19.0 (IBM Corp, Released 2010). The level of bilateral significance for all statistical tests was set at *α* = 0.05.

## Results

A total of 2.263 scientific documents were analyzed, accounting for a total of 27,432 citations, which indicated a ratio of citations per document of 12,12. For the sake of clarity and conciseness, only the main significant results are provided in Tables 2–3. Tables showing all comparisons can be provided upon request.

### Associations between scientific productivity and quality of mental healthcare

When examining Spearman’s rank-order correlation, significant associations were found between higher scientific production and higher quality of mental healthcare. In particular, higher research production (i.e., number of publications *p* = 0.022; *R*_s_ = 0.324, greater number of citations *p* = 0.036; *R*_s_ = 0.297, greater cumulative impact factor *p* = 0.023; *R*_s_ = 0.321, and higher Institutional H-Index *p* = 0.035; *R*_s_ = 0.298) were positively correlated with a higher proportion of nonreadmission discharges (i.e., individuals who were not subsequently readmitted to hospital after an initial discharge). In addition, higher research production (*p* = 0.021; *R*_s_ = −0.325), greater number of citations (*p* = 0.033; *R*_s_ = −0.302), greater cumulative impact factor (*p* = 0.022; *R*_s_ = −0.324), and higher H-Index (*p* = 0.034; *R*_s_ = −0.301) were also negatively associated with a lower readmission rate (see Table 1).

**Table 1.** Associations between indicators of scientific activity and measures of quality of mental healthcare.

	Research production		Number of citations		Cumulative impact factor		Institutional H-index	
	*Rs*	*p*	*Rs*	*p*	*Rs*	*p*	*Rs*	*p*
Nonreadmission discharges	0.324^a^	0.022	0.297^a^	0.036	0.321^a^	0.023	0.298^a^	0.035
Readmission rate	−0.325^a^	0.021	−0.302^a^	0.033	−0.324^a^	0.022	−0.301^a^	0.034
Proportion of referrals	0.290^a^	0.041	0.263	0.065	0.296^a^	0.037	0.259	0.069

a Significance was set at *α* = 0.05. Only significant results are presented (see results).

Quality of care variables were: nonreadmission discharges, length of stay (hospitalization), readmission rate, planned admissions, voluntary admissions, discharges to home, referrals, exitus, and nonreadmission discharges by diagnosis.

Research activity variables were: research production (number of publications), number of citations, ratio of citations per document, national collaborations, international collaborations, average impact factor, cumulative impact factor, and institutional H-index.

More specifically, higher scientific productivity in terms of higher research production (i.e., number of publications *p* = 0.026; *R*_s_ = 0.315, higher number of citations *p* = 0.041; *R*_s_ = 0. 290, higher cumulative impact factor *p* = 0.025; *R*_s_ = 0.317, and higher Institutional H-Index *p* = 0.036; *R*_s_ = 0.297) was positively correlated with a higher proportion of nonreadmission discharges in patients with schizophrenia**.** In addition, a significant positive association was found between higher research production (*p* = 0.006; *R*_s_ = 0.387), higher number of citations (*p* = 0.010; *R*_s_ = 0.363), higher cumulative impact factor (*p* = 0.010; *R*_s_ = 0.359), higher Institutional H-Index (*p* = 0.008; *R*_s_ = 0.373), and higher number of nonreadmission discharges in patients with alcohol dependence syndrome (see Table 2).

**Table 2.** Associations between indicators of scientific activity and nonreadmission discharges by diagnosis.

	Research Production		Number of citations		Ratio of citations per document		Cumulative impact factor		Institutional H-index	
	*Rs*	*p*	*Rs*	*p*	*Rs*	*p*	*Rs*	*p*	*Rs*	*p*
Schizophrenic disorders	0.315^a^	0.026	0.290^a^	0.041	0.041	0.776	0.317^a^	0.025	0.297^a^	0.036
Other nonorganic psychoses	0.269	0.058	0.268	0.060	0.137	0.343	0.279^a^	0.050	0.241	0.092
Cyclothymic disorder	−0.236	0.100	−0.273	0.055	−0.201	0.161	−0.266	0.062	−0.305^a^	0.031
Alcohol dependence syndrome	0.387^a^	0.006	0.363^a^	0.010	0.174	0.227	0.359^a^	0.010	0.373^a^	0.008
Generalized developmental disorders	0.182	0.206	0.219	0.126	0.304^a^	0.032	0.189	0.190	0.176	0.223

a Significance was set at *α* = 0.05. Only significant results are presented (see results).

Quality of care variables were: nonreadmission discharges, length of stay (hospitalization), readmission rate, planned admissions, voluntary admissions, discharges to home, referrals, exitus, and nonreadmission discharges by diagnosis.

Research activity variables were: Research production (number of publications), number of citations, ratio of citations per document, national collaborations, international collaborations, average impact factor, cumulative impact factor, and institutional H-index.

When the outcome variables where explored by diagnostic group, higher scientific activity, as measured by increased research production (*p* = 0.022; *R*_s_ = 0.322), higher number of citations (*p* = 0.040; *R*_s_ = 0.292), higher cumulative impact factor (*p* = .021; *R*_s_ = 0.325), and higher Institutional H-Index (*p* = 0.034; *R*_s_ = 0.301), were significantly associated with a higher proportion of nonreadmission discharges in patients with nonaffective psychosis (Table 3).

**Table 3.** Associations between indicators of scientific activity and nonreadmission discharges by diagnostic group.

	Research production		Number of citations		Cumulative impact factor		Institutional H-index	
	*Rs*	*p*	*Rs*	*p*	*Rs*	*p*	*Rs*	*p*
Nonaffective psychosis disorders	0.322^a^	0.022	0.292^a^	0.040	0.325^a^	0.021	0.301^a^	0.034
Developmental disorders	0.302^a^	0.033	0.253	0.076	0.309^a^	0.029	0.255	0.074

a Significance was set at *α* = 0.05. Only significant results are presented (see results).

Quality of care variables were: nonreadmission discharges, length of stay (hospitalization), readmission rate, planned admissions, voluntary admissions, discharges to home, referrals, exitus, and nonreadmission discharges by diagnosis.

Research activity variables were: research production (number of publications), number of citations, ratio of citations per document, national collaborations, international collaborations, average impact factor, cumulative impact factor, and institutional H-index.

### Scientific production predictors of quality of mental healthcare

Backward stepwise multiple regression models (controlling for number of beds and population, when appropriate) were used to determine the scientific production predictors of quality of mental healthcare delivered in the selected psychiatric units of the clinical research centers of interest. A total of 7 predictive models were built from the significant results obtained based on the reported association between variables of scientific activity and mental healthcare outcomes.

For the first model, proportion of number of documents (research production) was entered as the independent variable and explained a significant proportion of the change in readmission rate (*p* = 0.022; *R* = −0.324). Specifically, higher research production over time predicted a significant amount (8.6%) of the variance of change in readmission rate (decrease) when other variables were controlled for.

In the second model, a backward multiple regression was run to explore the effect of a higher cumulative impact factor (independent variable) on the prediction of an increase in the number of hospital referrals, controlling for the number of beds and population in the study. As a result, this independent variable statistically predicted 6.9% of the variance of the increase in referrals (*p* = 0.036; *R* = 0.297) when accounting for other variables in the model.

In the third model, the cumulative impact factor was entered as the independent variable and explained a significant proportion of change in nonreadmission discharges in patients with schizophrenic disorders (*p* = 0.025; *R* = 0.317). A higher cumulative impact factor predicted an 8.2% increase in the proportion of nonreadmission discharges in patients with schizophrenic disorder over time when accounting for the effect of the covariates.

In a fourth model, the proportion of number of documents (research production) and Institutional H-index were entered as predictive variables for the change over time in the proportion of nonreadmission discharges (dependent variable) in patients with cyclothymic disorder. Both production and Institutional H-index predicted a significant amount (17.4%) of the variance of change in nonreadmission discharges (increase) (*p* = 0.012, *R* = 0.456; (,) = *p* = 0.004, *R* = 0.456) when other variables in the model were accounted for.

For the fifth model, the proportion of number of documents (research production) was entered as the independent variable and explained a significant proportion of nonreadmission discharges in patients with alcohol dependence syndrome (*p* = 0.006; *R* = −0.387). Specifically, an increase in research production over time predicted a significant amount (13.2%) of the variance of change in nonreadmission discharges (increase) when controlling for other variables in the model.

In a sixth model, the ratio of citations per document was entered as the independent variable and explained a significant proportion of nonreadmission discharges in patients with generalized developmental disorders (*p* = 0.032; *R* = 0.304). An increase in this rate over time predicted a significant amount (7.3%) of the variance of change in nonreadmission discharges (increase) when controlling for covariates.

In the last model, a higher cumulative impact factor over time predicted a significant amount (8.7%) of the variance of change (increase) in the proportion of nonreadmission discharges in patients with nonaffective psychosis disorders (*p* = 0.021; *R* = −0.325) when all variables in the model were accounted for.

## Discussion

In the current study, we found an association between higher research productivity/outputs in leading clinical research centers and better quality of mental healthcare delivered in their psychiatric units. Specifically, our results suggest that the higher the scientific production, the lower the level of readmissions in certain diagnoses and the readmission rates, reflecting maintained improvement in the patient condition, obviating the need for further hospital admission. These findings may indicate that, in centers with high research activity, patients have better healthcare and, therefore, less chance of readmission once discharged.

Although a few studies have found significant correlations between research data and quality-of-care outcomes [5,6], the results of the present study systematically denote fewer readmissions for schizophrenic disorders, neurodevelopmental disorders, and nonaffective psychoses in centers with high research activity. Furthermore, research activity measures had a predictive value for nonreadmission discharges in patients with these disorders, which might indicate that it is possible to determine which centers will provide more stable recovery over time, once patients with these conditions are discharged from inpatient units. Similarly, centers with higher scientific activity have a lower level of readmissions in patients with alcohol dependence syndrome, and research production also has a predictive value for nonreadmission discharges with this diagnosis. The results obtained in patients with developmental disorders are particularly striking, as they might show that higher research activity predicts a lower number of readmissions in patients with this diagnosis. There is little training in hospitalization units for this type of neurodevelopmental disorder, which also includes autism, so high research activity in these conditions suggests that lower readmission rates may be related to a higher level of clinical specialization in this type of disorder.

In contrast, patients with cyclothymic disorder tend to have a higher proportion of readmissions to centers with more research activity. The predictive value associated with a higher proportion of nonreadmission discharges in patients with this condition is related to higher productivity and less impact on research. Contrary to our main hypothesis, this could be related to the specific population enrolled in our current study and the existence of specific clinical programs for mood disorders in hospitals with higher scientific productivity (e.g., the Bipolar Program at Hospital Clinic in Barcelona) to which patients are referred from a particular clinical catchment area. Thus, patients with particularly severe and recurrent mental illness are admitted to these programs at the largest/most productive hospitals (the ones analyzed in the current study), referred by community services, which in turn handle the less severe clinical cases. That is to say, it is plausible to find that most productive centers provide better healthcare if only their specific catchment area/referral population is considered. However, if these clinical centers are referral hospitals for a specific mental health condition (e.g., cyclothymic disorder), they would probably be receiving referrals for the most severe cases in the population, which can result in selection bias. Future research on this matter is warranted.

As for the results obtained in the regression analyses, these point in the direction of the main hypothesis of the study. Although the predictive value found between the measures of scientific activity and quality of care is generally low, increased research measures may be associated with better health outcomes.

Limitations of the current study include the exclusion of psychology and the sources of information used (only data from the Web of Science). This was an exploratory study. Thus, corrections for multiple comparisons were not done, which could be perceived as a limitation. Since multiple testing adjustments control false positives at the potential expense of false negatives, we chose to report all comparisons (*p*-values) and regard our findings as tentative [10,11]. These limitations notwithstanding, the current study suggests a relationship between research outputs and clinical outcomes. Future studies should pursue this line of research by expanding it to other scientific areas, complementing quality-of-care data with indices of users’ subjective satisfaction, increasing the number of indicators of both quality of care and scientific activity and including more sources of information such as PubMed, Scopus, and/or Google Scholar and/or including data from other countries.

Our results may inform health institutions and public and private funding agencies of relevant research measures that could facilitate the decision-making involved in clinical and research management. This would allow appropriate distribution of funding to health managers, resulting in more effective hiring of specialized clinical research staff, establishing the best indicators for career advancement, promoting research training among residents, development of more accurate research and clinical policies and quality-of-care assessment processes within psychiatric units and a resulting improvement in a patient-based perspective, and for the prognosis of individuals with psychiatric conditions.

## Data Availability

The data that support the findings of this study are available from Health Information Institute from The Spanish Ministry of Health. Restrictions apply to the availability of these data, which were used under request for this study. Data are available from the authors with the permission of Health Information Institute from The Spanish Ministry of Health.
